# PLC-beta 1 regulates the expression of miR-210 during mithramycin-mediated erythroid differentiation in K562 cells

**DOI:** 10.18632/oncotarget.1972

**Published:** 2014-05-14

**Authors:** Alberto Bavelloni, Alessandro Poli, Roberta Fiume, William Blalock, Alessandro Matteucci, Giulia Ramazzotti, James A. McCubrey, Lucio Cocco, Irene Faenza

**Affiliations:** ^1^ SC Laboratory of Musculoskeletal Cell Biology, Rizzoli Orthopedic Institute, Bologna, Italy; ^2^ Laboratory RAMSES, Rizzoli Orthopedic Institute, Bologna, Italy; ^3^ Cell Signaling Laboratory, Department of Biomedical Sciences, University of Bologna, Bologna, Italy; ^4^ CNR-National Research Council of Italy, Institute of Molecular Genetics, Bologna, Italy; ^5^ Department of Microbiology & Immunology, Brody School of Medicine, East Carolina University, Greenville, NC, USA

**Keywords:** phospholipase Cβ1, erythropoiesis, K562, miR-210, γ-globin

## Abstract

PLC-beta 1 (PLCβ1) inhibits in human K562 cells erythroid differentiation induced by mithramycin (MTH) by targeting miR-210 expression. Inhibition of miR-210 affects the erythroid differentiation pathway and it occurs to a greater extent in MTH-treated cells. Overexpression of PLCβ1 suppresses the differentiation of K562 elicited by MTH as demonstrated by the absence of γ-globin expression. Inhibition of PLCβ1 expression is capable to promote the differentiation process leading to a recovery of γ-globin gene even in the absence of MTH. Our experimental evidences suggest that PLCβ1 signaling regulates erythropoiesis through miR-210. Indeed overexpression of PLCβ1 leads to a decrease of miR-210 expression after MTH treatment. Moreover miR-210 is up-regulated when PLCβ1 expression is down-regulated. When we silenced PKCα by RNAi technique, we found a decrease in miR-210 and γ-globin expression levels, which led to a severe slowdown of cell differentiation in K562 cells and these effects were the same encountered in cells overexpressing PLCβ1. Therefore we suggest a novel role for PLCβ1 in regulating miR-210 and our data hint at the fact that, in human K562 erythroleukemia cells, the modulation of PLCβ1 expression is able to exert an impairment of normal erythropoiesis as assessed by γ-globin expression.

## INTRODUCTION

MicroRNAs (miRNAs) are small regulatory RNAs that regulate gene expression post-transcriptionally, including both inhibition of protein translation and mRNA degradation [1]. Indeed miRNAs are involved in the regulation of a variety of biological processes such as embryonic development, cell proliferation, and differentiation [2]. Altered expressions of miRNAs have been implicated in hematological malignancies [3]. In many cases, the identification of specific targets of miRNAs highlighted important cellular events and demonstrated that aberrant microRNA expression is a common feature of many diseases [4]. Several miRNAs were identified as deeply involved in the erythroid phenotype, including miR-15a, miR-16-1, miR-126, miR-144, miR-451 and miR-210 are believed to regulate several functions of erythroid cells such as maturation and proliferation of early erythroid cells, expression of fetal γ-globin genes and enucleation [5]. Recent studies have shown that dysregulation of miRNAs contributes to the initiation, progression, metastasis, and drug resistance of cancer [6] and hematologic malignancy [7]. Previously, several miRNAs have been identified as being involved in hematopoiesis through their target genes. Moreover, miR-223 enhances retinoic acid (RA)-induced granulocytic differentiation by targeting nuclear factor I/A (NFIA) [8]. In addition MiR-155 transduction greatly reduces both the myeloid and erythroid colony formation of normal human hematopoietic stem-progenitor cells [9], miR-221 and miR-222 inhibit normal erythropoiesis and erythroleukemia cell growth via kit protein down-regulation [10], miR-451 and GATA transcription factors were shown to comprise a regulatory circuit that modulates erythroid maturation [11-13], miR-210 is involved in increased expression of the γ-globin gene in differentiating erythroid cells [14]. K562, a myelogenous leukemia cell line arising from a highly undifferentiated progenitor of the erythrocytic and megakaryocytic lineages [15] with potential to megakaryocyte and erythroid differentiation, provides an excellent model system for investigating processes involved in cellular differentiation [16]. MiR-210 has been mechanistically linked to the control of a wide range of cellular responses known to influence normal cell developmental as well as a number of hypoxia-dependent disease states, including tissue ischemia, inflammation, and tumorogenesis [17]. Recently it has been described that miR-210 is a highly expressed miR in the erythroid precursor cells and is induced during MTH mediated erythroid differentiation of K562. MTH is a powerful inducer of erythroid differentiation and γ-globin gene expression in erythroid cells and namely of K562 erythroid differentiation, leading to accumulation of γ-globin mRNA and production of embryo-fetal haemoglobins [14,18]. The understanding of the regulatory mechanism of erythroid differentiation is of crucial importance in order to develop methods for the manipulation of hematopoietic stem cells suitable for the treatment of anemia. Our previous studies have shown that Phospholipase C β1 (PLCβ1) is a key molecule for nuclear inositide signaling, where it plays a role in cell cycle progression, proliferation and differentiation [19-23]. PLCβ1 is responsible for the hydrolysis of the phosphatidilinool-4,5-bisphophate (PIP2), generating the second messengers inositol-1,4,5-triphosphate and diacylglycerol. Nuclear PI metabolism changes during dimethyl sulfoxide (DMSO)-induced erythroid differentiation of FELC [24] and the nuclear localization of PLCβ1 is crucial for the differentiation of FELC in that PLCβ1 signaling targets the transcription factor p45/NF-E2, an enhancer binding protein for β-globin gene, whilst other transcription factors involved in erythroid differentiation, such as members of the GATA family, do not respond to nuclear PLCβ1 signaling [25]. Another nuclear PLC-β1 target identified by a proteomic approach is SRp20, a splicing factor that belongs to the serine/arginine-rich (SR) splicing factor family. PLC-β1 and SRp20 physically interact in the nucleus and SRp20 expression is down-regulated by the nuclear signaling evoked by PLC-β1 [26]. In addition, nuclear PLCβ1 up-regulates the expression of CD24 in MEL cells. CD24 is an antigen involved in differentiation and hematopoiesis, it is considered a critical molecule for the metastasizing ability of solid tumors and it is overexpressed in a number of leukemias. Because of these assumptions we decided to investigate whether the PLCβ1 signaling pathway could be involved in MTH-induced erythroid differentiation of K562 cells and to examine whether PLCβ1 regulates miR-210 expression. Our results provide experimental support for the idea that PLCβ1 is involved in the control of erythroid differentiation by means of the regulation of miR-210 expression, likely through a PKCα-mediated-pathway.

## RESULTS

### Characterization of PLCβ1 and γ-globin expression in erythroid cell culture

**Fig 1:**
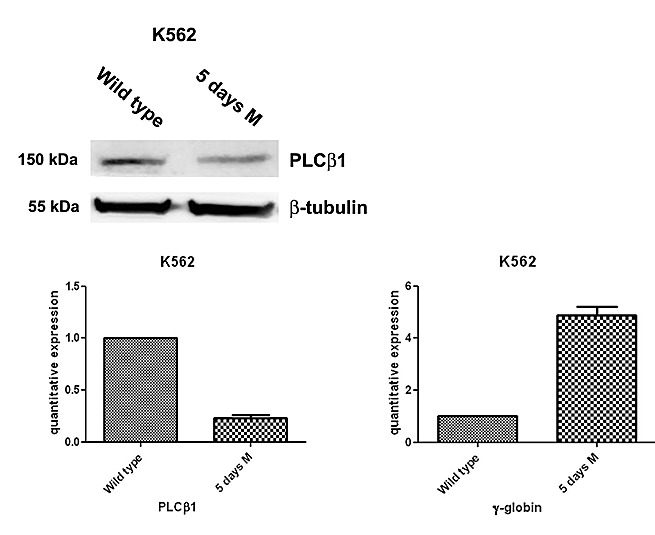
PLCβ1 expression decreases during erythroid MTH induction of human K562 cells Cells were grown in complete RPMI 10% FBS for two days and, then, divided in two aliquots: one was seeded in complete RPMI 10% FBS and the other one in complete RPMI 10% FBS supplemented with mithramaycin (MTH) to a final concentration of 30 nM. K562 cells were harvested after 5 days of growth in proliferating (wild type) or differentiation medium (5 days M). Cells were then lysed to extract RNA and proteins. Western blotting analyses were carried out with specific antibodies against PLCβ1 and β-tubulin. The extracted RNA was retro-transcribed and real-time PCR was performed to evaluate the quantitative expression of PLCβ1 and γ-globin. As shown, PLCβ1 highly decreases after 5 days of differentiation both in Western Blotting and in real-time PCR. γ-globin expression was used to check the differentiation of K562 cells.

K562 cells can be induced to differentiate towards erythroid lineage by MHT. MHT is a highly glycosylated aureolic acid anticancer agent [27] a well-known inducer of K562 cells. In a previous study aimed at elucidating the functional role of PLCβ1 in the regulation of growth and differentiation of Friend erythroleukemia cells, we overexpressed PLCβ1 and we demonstrated that this overexpression was sufficient to block erythroid differentiation induced by DMSO and, therefore, to induce cell cycle progression. Thus, we analyzed whether PLCβ1 was present in K562 cell line. We performed western blot analysis using a specific antibody against PLCβ1. As we show in fig.1 our antibody recognize this PLC in whole cell extract. Then we analyze the presence of PLCβ1 during erythroid differentiation and we observed that PLCβ1 content was decreased in MTH-treated K562 cells as expected. β-tubulin was used as loading control. To validate at the transcriptional level the data obtained by western blot analysis, cells from the same samples were used for mRNA extraction and cDNA synthesis for real-time PCR. PLCβ1 mRNA level decreased after induction of differentiation in respect to protein expression data. Moreover, the effect of MTH on the mRNA levels of γ-globin in K562 cultures was determined by quantitative RT-PCR. The cells were induced to erythroid differentiation by 30 nM MTH. Five days after 30nM MTH treatment K562 cells showed signs of erythroid differentiation. By this time γ-globin is expressed at a very high level showing that the majority of the cells are committed to the differentiation program. In fact, by comparison, MTH stimulated γ-globin expression increased by about five fold, compared to the same day control throughout the time period of the experiment. This suggested that MTH was able to induce K562 cells towards erythroid differentiation along with the elevated expression of γ-globin. In agreement with the known behavior of nuclear inositide signaling [28] five days of erythroid differentiation largely decreases the expression of PLCβ1 as shown in fig. 1. Taken together, the present data demonstrates that MTH suppressed expression of PLCβ1 during K562 differentiation program, suggesting an important role for this protein in the regulation of erythropoiesis.

### Effect of PLCβ1 silencing and overexpression on MTH induced K562 differentiation

**Fig 2:**
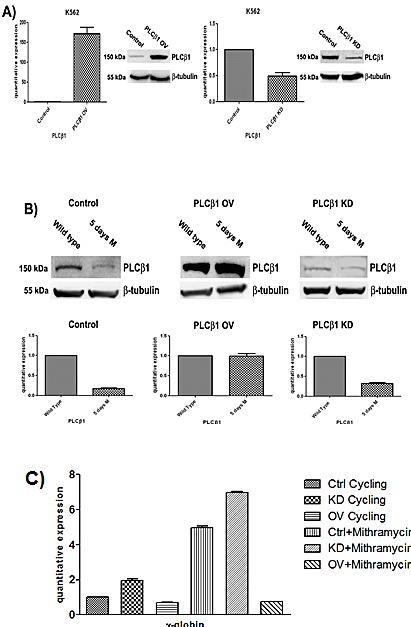
Effect of PLCβ1 modulation on the MTH erythroid differentiation of K562 cells A) Clones were analyzed by Western Blotting and Real-Time PCR to check the efficiency of PLCβ1 gene silencing (PLCβ1 KD) and the efficiency of PLCβ1 protein overexpression (PLCβ1 OV) respect to the controls (control). As shown PLCβ1 is respectively overexpressed and silenced in PLCβ1 OV and PLCβ1 KD if compared to control cells. B) Clones were grown in complete RPMI 10% FBS for two days and seeded in complete RPMI 10% FBS or in complete RPMI 10% FBS supplemented with mithramaycin to a final concentration of 30 nM. Cycling cells (wild type) and differentiating cells were harvested after 5 days of growth respectively in proliferating or differentiating condition (5 days M). Cells were then lysed to extract RNA and proteins. Proteins lysates were loaded at the same volume and protein concentration on an 8% SDS-PAGE gel. Western blotting analyses were carried out with specific antibodies against PLCβ1 and β-tubulin. The extracted RNA was retro-transcribed and real-time PCR was performed to evaluate the quantitative expression of PLCβ1. C) Clones were treated as in b) and γ-globin quantitative expression was evaluated by real-time PCR analysis. All results are representative of two different experiments using three separate clones of each sample.

To determine the functional role triggered by PLCβ1 during the erythroid differentiation, we performed expression experiments in wild type K562 cells, in cells in which PLCβ1 was overexpressed and in cells in which the PLCβ1 expression was knocked-down. The analysis of the PLCβ1 phenotype in the stable transfectants we obtained is shown in fig. 2A. The western blot analysis of these clones shows that we have obtained effectively stable clones of K562. mRNA was extracted, retro transcribed, and quantified by real-time PCR. Analysis of the PLCβ1 mRNA levels showed that both silencing and overexpressing actually occurred, in agreement with the variation of protein level shown in Fig. 2A. An analysis of the effect of differentiation program induced by MTH on PLCβ1 phenotype in the stable transfectants we obtained is shown in Fig. 2B. Cells transfected with the empty vector (mock) behave exactly as the original untransfected K562 cells do, in that PLCβ1, is down-regulated upon treatment with MTH. The same panel shows that when overexpressed, PLCβ1, is no longer down-regulated indicating that the overexpression of PLCβ1 determines a resistance to MTH treatment. Moreover the PLCβ1 KD shows an almost complete loss of expression both in the absence and in the presence of MTH, as expected. Hemoglobinization induction of K562 cells was efficient after MTH treatment. Erythroid differentiation was monitored using analysis of γ-globin transcripts by qRT-PCR. As shown in Fig. 2C, differentiation of wild-type K562 is characterized by activation of γ-globin gene expression. Moreover, transcription levels of γ-globin were elevated during MTH induced erythroid differentiation, but we observed a decrease of hemoglobinization in the samples in which PLCβ1 was overexpressed even in the presence of MTH. Further, cellular knockdown of PLCβ1 results in an increase of γ-globin expression of untreated cells but the highest level of hemoglobinization is observed after MTH treatment. Nevertheless, these findings provide a clear evidence of the critical involvement of the PLCβ1 in the differentiation process of MTH induced K562 cells.

### Expression of miR-210 in MTH-treated K562

**Fig 3:**
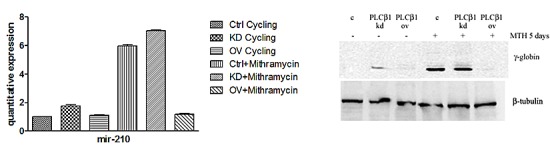
Effect of PLCβ1 expression on miRNA-210 after MTH treatment a) The figure shows the fold expression of the analyzed miR-210 in K562 control cells and in cells in which the expression of PLCβ1 is modulated. K562 cells were induced to differentiate with 30nM of MTH for 5 days. Real-Time PCR analysis of the expression of mir-210 was performed using let-7c expression as housekeeping control. b) Stably transfected K562 cells were differentiated and the level of differentiation was analyzed by γ-globin expression in the presence and in the absence of MTH. All results are representative of two different experiments using three separate clones of each sample.

**Fig 4:**
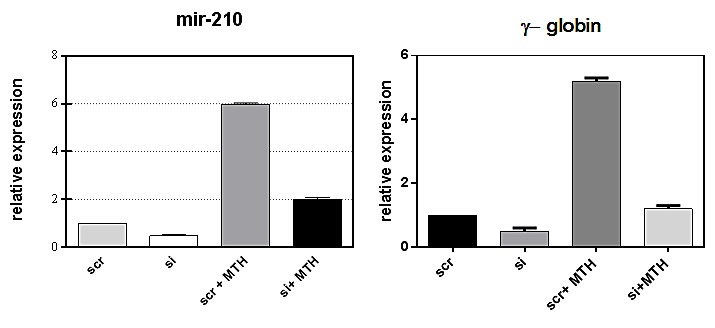
Loss of function study of miR-210 Inhibition of miR-210 activity by using anti-miR-210 treatment in K562 cells showed a significant depletion of miRNA-210 level as well as globin expression. K562 cells were induced to differentiate with 30nM of MTH for 5 days. The mean and standard deviation values were from 3 independent experiments.

Recent data identify a novel miR-210, whose expression is enhanced in association with erythroid differentiation and induction to fetal hemoglobin (HbF) production. The aim of this study was to investigate the PLCβ1 effect on miRNA-210 expression after MTH treatment. We employed both growing and differentiating cells harvested 5 days after MTH stimulation. Fig. 3A shows the expression of the analyzed miR-210 when RNA from K562 cells treated for 5 days with MTH is compared to that isolated from control untreated cells. Expression of the miRNA-210 in MTH-treated cells was determined by qRT-PCR. As it is clearly appreciable, during erythroid induction of K562 cells miR-210 expression increases. Then we analyzed the effects of overexpression and the effect of loss of function of this enzyme in K562 cells after 5 days of MTH administration. In proliferating K562 cells, cells transfected with PLCβ1 show no significant difference in the level of miR-210 compared to vector transfected cells. On the other hand, K562 cells silenced for the expression of the PLCβ1 show an increase of miR-210 levels as compared to control cells. The level of endogenous miR-210 in K562 control cells cultured for five days with MTH was increased as we expected. As it is clearly appreciable, during erythroid induction of PLCβ1 overexpressing K562 cells we assist to a dramatic decrease of miR-210 expression. Moreover, PLCβ1 silencing resulted in an increase in miR-210 expression after 5 days of MTH administration, even if only slightly, as compared with wild type differentiating cells. To examine the effect of modulated levels of PLCβ1 on erythroid differentiation of K562 cells, we evaluated, in the same samples, the expression of γ-globin. Western blot analysis (Fig. 3b) showed that amplified levels of PLCβ1 act to negatively regulate γ-globin protein levels after MTH treatment. On the contrary, the knockdown of PLCβ1 was able to increase the expression of γ-globin in K562 cells as compared with control cells even in the absence of the differentiating agent MTH. To investigate the contribution of miRNA-210 to erythroid cell differentiation under MTH treatment, loss of function study was performed using the anti-miR-210. The result showed that transfection of K562 cells with 10nM anti-miR-210 caused a significant reduction of miR-210 levels (Fig. 4). Determination of γ-globin expression in the presence of anti-miR-210 showed a partial but significant reduction of γ-globin mRNA levels under the MTH treatment at 72 h. Transfection with scramble (scr) caused no effect. Taken together, these results thus suggest that PLCβ1 signaling is involved in the erythroid differentiation process as assessed by γ-globin induced by MTH through miR-210 regulation and that this process is likely dependent on the amount of PLCβ1 expression level.

### PLCβ1 regulates miR-210 through PKCα

**Fig 5:**
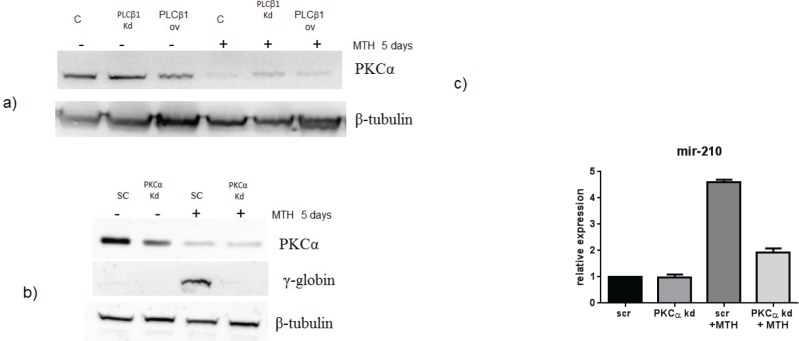
PKCα levels decrease in K562 cells overexpressing PLCβ1 a) Cell lysates were loaded at the same volume and protein concentration on a SDS-PAGE 10% gel, and western blotting analyses were performed with specific antibodies for PKCα. As shown, PKCα was characterized by a down modulation of its expression in cells overexpressing PLCβ1 in the presence and also in the absence of MTH; b) PKCα knockdown: K562 cells were transiently transfected with a specific siRNA to knockdown PKCα (PKCα) or with a scrambled (scr) one as negative control. Western blotting analyses were performed with specific antibodies for PKCα, γ-globin and β-tubulin. c) The figure shows the fold expression of the analyzed miR-210 in K562 control cells and in cells in which the expression of PKCα is knocked-down in presence and absence of MTH. Real-Time PCR analysis of the expression of mir-210 was performed using let-7c expression as housekeeping control. Results were the mean from three separate experiments ± SD.

In order to further investigate the pathway through which PLCβ1 could modulate miR-210 and cell differentiation in K562 cells, we investigated the possible role of PKCs, known to be direct targets of PLC signaling. Overexpression of PLCβ1 led to a strong decrease in PKCα levels if compared with the controls (Fig. 5a); meanwhile, PKCβII and PKCζ levels were not affected at all (data not shown and ref. 39). These data showed a possible connection between PLCβ1, PKCα and miR-210 modulation in K562 cell differentiation. Then, we transiently transfected K562 cells to silence PKCα through RNAi technique. Cells were transfected with specific siRNA for PKCα and scrambled one as control. Twenty-four hours after the transfection, we induced K562 differentiation with MTH for 5 days and the expression of both miR-210 and γ-globin have been analyzed. Interestingly, as reported in Fig. 5b, cells characterized by silenced PKCα showed a significant decrease of miR-210 and γ-globin expression if compared with the one transfected with scrambled siRNA. Moreover to examine the effect of decreased levels of PKCα on erythroid differentiation, we evaluated, in the same samples, the expression of miR-210. As can be seen in Fig. 5c, miR-210 levels increased in differentiated cells, but PKCα knock-down cells did not show increase of miR-210 levels. These data confirmed that PLCβ1 can regulate miR-210 levels through PKCα signaling pathway.

## DISCUSSION

In this study, we used human erythroleukemia K562 cells as an in vitro model of erythropoiesis because they have been proven to be a powerful tool for investigating erythroid cell development. To address the functional relevance of PLCβ1 in erythropoiesis, we have modulated PLCβ1 expression into MTH treated K562 cells and studied its effect on erythroid differentiation and miR-210 profile. The DNA binding drug MTH is a potent inducer of γ-globin mRNA accumulation and fetal hemoglobin production in erythroid cells from healthy human subjects and β-thalassemia patients [18]. MiR-210 is an important microRNA target that has been demonstrated to be associated with MTH-mediated induction of erythroid differentiation of leukemic K562 cells and HbF production in erythroid precursor cells from β-thalassemia patients [14]. In fact, Bianchi et al. demonstrated that MTH is a powerful inducer of erythroid differentiation and γ-globin gene expression in human erythroid cells. Our results suggest that PLCβ1 signaling is involved in the differentiation process induced by MTH by regulating both γ-globin and miR-210 expression. Indeed PLCβ1 overexpression inhibits erythroid differentiation of the erythroid lineage K562 cell line. Therefore, cellular knockdown of PLCβ1 causes a significant up-regulation of γ-globin expression. This indicates that the level of expression of PLCβ1 is able to affect the erythroid potential of these cells. In the current study we identified miR-210 as a target of PLCβ1. In fact we observed that PLCβ1 overexpression led to a loss of induction of miR-210 expression after MTH treatment. Moreover, K562 cells silencing for the expression of the PLCβ1 induces an increase of miR-210 levels. Enhanced PLCβ1 level and reduced miR-210 level accompanies erythroid differentiation. This suggest that the role of PLCβ1 during erythropoiesis is linked to that of miR-210 even though our results do not fully explain the mechanism of action of PLCβ1 within the cells but the results obtained are indeed compatible with previously results that demonstrated a role for PLCβ1 in erythropoiesis [24,25]. We also showed that inhibition of miR-210 caused the significant reduction of globin gene expression in K562 erythroid cell line. Interestingly, we found that PKCα levels decreased in cells where PLCβ1 was overexpressed. All in all, the data presented in this study suggest that an enhanced PLCβ1 signaling could lead, through generation of second messengers, to a sustained activation of PKCα and then to its degradation. At any rate, the mechanism that links these two proteins will be further investigated in the future. When we silenced PKCα by RNAi technique, we found a decrease in miR-210 and γ-globin expression levels, which led to a severe slowdown of cell differentiation in K562 cells and these effects were the same encountered in cells overexpressing PLCβ1. Hematopoiesis is a multistep process leading to the production of mature blood cells, and is controlled by many factors, including miRNAs. MiR-210 is a member of a new class of regulatory miRNAs that play an important role in erythroid maturation [29]. MiR-210 is induced in time-dependent and dose dependent manner during MTH-mediated erythroid induction of K562 cells. Moreover, miR-210 is induced following MTH-treatment also in erythroid precursor cells from human donors [14]. MiR-210 has been recently associated with hypoxia [30-33]. MiR-210 was also found up-regulated in most solid tumors and its expression correlates with an adverse clinical outcome and with metastatic potential [34,35]. In keeping with these data, miR-210 has been proposed as a novel tumor hypoxia marker [36]. Recently it has been demonstrated that Pancreatic Stellate cells induce the expression of miR-210 in pancreatic cancer cells [37]. In this cellular model MiR-210 up-regulation is inhibited by inhibitors of ERK and PI3K/Akt pathways. It was already known that PLCβ1 is down-regulated when Friend erythroleukemia cells treated with DMSO differentiate and synthesize β-globin [38]. Another study on the effects of the overexpression of PLCβ1 showed a specific and positive connection between cyclin D3 and PLCβ1 in K562 cells, which led to a prolonged S phase of the cell cycle and a delay in cell proliferation in that PLCβ1 targets cyclin D3, likely through a PKCα-mediated-pathway and, as a downstream effect of its activity, K562 cells undergo an accumulation in the S phase of the cell cycle [39]. PLCβ1 interacts with a number of proteins involved in cellular processes, and in different experimental models [20,40]. Very recently Scarlata et al. have shown that PLCβ1 can affect the siRNA activity of genes for two metabolic enzymes, GAPDH and LDH, through its interaction with TRAX (translin-associated factor X) [41] which is a strong binding factor of PLCβ1 [42,43]. To find out new PLCβ1 molecular targets, different from proteins, such as miRNA could give rise to new and unexpected findings that are important for understanding the role of this pathway in physiology and pathology as well as in the mode of action of DNA binding drugs such as MTH which is a potent inducer of γ-globin mRNA accumulation and HbF production in erythroid cells from healthy human subjects and β-thalassemia patients [18]. On the whole our data hint at a new pathway leading to erythroid differentiation that links PLCβ1 signaling with the expression of miR-210 and eventually of γ-globin.

## MATERIALS AND METHODS

### Culture conditions

Human leukemia K562 cells were cultured in humified atmosphere of 5% CO2/air in RPMI 1640 medium (Sigma, St Louis, MO, USA), 10% fetal bovine serum (Sigma Chemical St Louis, MO). To induce erythroid differentiation the cells (10^6^/mL) were treated with 30nM mithramycin (Sigma Chemical St Louis, MO) for 3 or 5 days.

Transfections and isolation of clones. Cells were transfected with full-length DNA vectors for human PLCβ1a and cloned into pcDNA/2.1 plasmids. Overexpression of PLCβ1 was performed using Lipofectamine 2000 from Invitrogen. Cells were seeded at a cell density of 5*105/ml in 25ml flasks in which was added the mix of Lipofectamine 2000 and the right vectors, following manufacturer's instructions. Cells were collected to be analyzed 24h after the transfections. To obtain stable clones, cells were selected by limiting dilution in complete RPMI 1640 10% FBS containing Geneticin (G418 from Sigma Aldrich) at a concentration of 1000μg/ml 24h after the transfection, then expanded and kept always in selection with G418. The expression of PLCβ1 was silenced using RNAi techniques with the electroporation assay kit by Thema Ricerca. Cells, plated at a cell density of 5*10^5^/ml before the transfection, were resuspended in 100μl of Mirrus Solution (pre-warmed at 37°C); specific siRNAs were added at the suspension and the mix moved in proper cuvettes. Using program T-16, cells were electroporated in Nucleofector I (Amaxa). Afterwards, cells were plated in 25ml flasks with 5ml of complete RPMI 1640 10% FBS. The following siRNA was used at a concentration of 50nM: for PLCβ1 (Ambion) and as negative control (scrambled), S00J1C8 (Ambion). PKCα Knock-down: the expression of PKCα was silenced using RNAi techniques with the electroporation assay kit by Thema Ricerca. Cells, plated at a cell density of 5 × 10^5^/ml before the transfection, were resuspended in 100 μl of Mirrus solution (prewarmed at 37°C); specific siRNAs were added at the suspension and the mix moved in proper cuvettes. Using program T-16, cells were electroporated in Nucleofector I (Amaxa). Afterwards, cells were plated in 25 ml flasks with 5 ml of complete RPMI 1640 10% FBS. The following RNAis were used at a concentration of 50 nM: for PKCα, PRKCA s11092 (Ambion) and as negative control (scrambled), S00J1C8 (Ambion).

### RNA isolation and MicroRNA analysis

Total RNA was isolated by miRVana miRNA isolation Kit (Ambion by life technology) according to the manufacture instructions. The Nanodrop2000 UV-Vis Spectrophotometers was used to determine the integrity and measure the concentration of total RNA samples (Thermo Scientific Inc). For microRNA quantization reverse transcriptase reactions were performed using TaqMan MicroRNA Revese Transcription Kit (Applied Biosystems for hsa-miR-210 and the 7700 Sequence Detection System version 1.7 (Applied Biosystems, Foster City, CA, USA). Relative expression was calculated using the comparative cycle threshold method and as reference genes the endogenous control human 18S kit human 18S rRNA and hsa-let-7c microRNA. Anti-miR-210 inhibitor transfection: anti-miR™ miRNA inhibitor (Applied Biosystems, Foster City, CA), single-stranded RNA-based inhibitor was used for inhibition of miRNA activity. MiR-210 inhibitor (anti-miR-210) or negative control molecules (scrambled oligonucleotide) were transfected into K562 cells or day 7 erythroid precursor cells using Lipofectamine 2000 (Invitrogen Carlsbad, CA) at a final concentration of 10 nM.

### Western Blotting

Approximately 2.5 × 10^6^ cells were harvested at indicated time points, washed twice in PBS, resuspended in RIPA lysis buffer (50 mM Tris-HCl at pH 7.4, 150 mM NaCl, 0.1% SDS, 1% NP-40, 0.25% sodium deoxycholate, protease inhibitors coktail (Roche)), and incubated for 10 min on ice. After centrifugation at 14,000 rpm for 5 min at 4°C to remove cellular debris, the remaining supernatant was transferred to a new tube, supplemented with sample buffer, and incubated for 10 min at 60°C. Proteins were separated by SDS gel electrophoresis using the NuPAGE Bis-Tris gel system (Invitrogen) and MOPS running buffer under reducing conditions. Subsequently, proteins were transferred onto a nitrocellulose membrane using the NuPAGE transfer buffer (Invitrogen). Membranes were blocked with 5% milk-PBST for at least 1 h and probed with PLCβ1 rabbit polyclonal antibody (R-233; sc-9050, Santa Cruz Biotechnology) at a 1:500 dilution, or β-tubulin mouse monoclonal from Sigma-Aldrich at a 1:10000 dilution in PBST for 1 h at room temperature or overnight at 4°C. PKCα was from Cell Signaling Technology). Membranes were washed four times with PBST, incubated with sheep anti-mouse or anti-rabbit peroxidase-coupled secondary antibodies (NA931 and NA934, GE Healthcare) at a 1:2000 dilution in 2.5% milk-PBST for 1 h at room temperature, washed three times with PBST, and incubated for 2 min with Western Lightning Plus-ECL substrate (Perkin Elmer). Proteins were visualized by exposure to scientific imaging film (Kodak).

### Quantitative RT–PCR

Isolation of RNA was performed using the miRNeasy minikit (Qiagen). RNA was quantified by a NanoDrop spectrophotometer (Thermo Scientific). Reverse transcription was carried out using the iScript cDNA synthesis kit (Bio-Rad). Real-time PCR was performed using the ABI 7900 Machine Real-Time PCR system and SYBR Green PCR Master Mix (Applied Biosystems). To determine PLCβ1 and γ-globin expression, the samples used for the reporter gene assay were harvested, and total cellular RNA was extracted using the RNeasy mini kit (Qiagen, Hilden, Germany) according to the manufacturer's instructions. After the measurement of the RNA concentration, cDNA was synthesized starting from 2 μg of total RNA using 200 U of M-MLV retrotranscriptase (Promega, Madison, WI, USA), 0.5 μg of oligo-dT primers, 25 U ribonuclease inhibitor, and 10 mM of each dNTP. The reactions were incubated for 1 h at 42°C. The expression of PLCβ1 and γ-globin genes was determined by using a TaqMan-based real-time PCR method (Applied Biosystems, Foster City, CA, USA). Their expression levels were analyzed and quantified by means of TaqMan-specific probes Hs01008373 for PLCβ1 (Applied Biosystems); Hs00361131_g1 for γ-globin. GAPDH was used as the reference housekeeping gene (Mm99999915_g1; Applied Biosystems). All real-time PCR reactions were performed in a MicroAmp Optical 96-well reaction plate (Applied Biosystems) in a total reaction volume of 25 μl, using 12.5 μl of TaqMan PCR universal master mix (Applied Biosystems), 1.25 μl of the desired gene expression assay mix containing the primers and probes, and 1 μl of cDNA. Each reaction was repeated in triplicate. Reactions were carried out using the ABI Prism 7300 sequence detection system (Applied Biosystems) with the following thermal conditions: 50°C for 2 min and 95°C for 10 min, followed by 40 cycles of 95°C for 15 s, and 60°C for 1 min. The ΔΔ*C_t_* method was used to quantitate amounts of each gene relative to the GAPDH amount in each reaction, according to the manufacturer's protocol (Applied Biosystems). The results of different sets of experiments were statistically analyzed by GraphPad Prism 3.02 software (GraphPad, San Diego, CA, USA).
